# A Partial Discharge Detection Approach in Distribution Cabinets Using a Mach–Zehnder Interferometer

**DOI:** 10.3390/s25072265

**Published:** 2025-04-03

**Authors:** Junliang Wang, Ying Zhang, Xiang Gu

**Affiliations:** 1 School of Microelectronics, Nanjing University of Science and Technology, Nanjing 210094, China; zhying@njust.edu.cn; 2 CMEC Comtrans International Co., Ltd., Beijing 100055, China; guxiang@cmec.com

**Keywords:** distribution cabinets, Mach–Zehnder interferometer, partial discharge, ultrasonic waves

## Abstract

Distribution cabinets are of paramount importance in power supply systems. Internal partial discharge may result in power interruption or even the outbreak of fire. This paper proposes a partial discharge (PD) detection approach based on a fiber-optic Mach–Zehnder interferometer (MZI). The MZI method utilizes a fiber wound with a certain size and number of turns as the sensing element, which is mounted on the wall of the low-voltage distribution cabinet to monitor the partial discharge within the cabinet in real time. A true-type distribution cabinet partial discharge experimental platform is developed to validate the proposed method. Three 10 m long fiber-optic sensors with diameters of 50 mm, 80 mm, and 100 mm are designed and compared with a traditional piezoelectric transducer (PZT) for analysis. The experimental results indicate that the fiber-optic MZI sensor can effectively capture PD acoustic pulses, and the pulse amplitude is consistent with that of the PZT sensor. Moreover, compared with the PZT sensor, the fiber-optic MZI system possesses a higher frequency response and a longer effective detection time for PD pulses, demonstrating superior PD detection performance. The fiber-optic coil sensor with a diameter of 8 cm performed optimally in the experiment. The fiber-optic sensing method based on the MZI has significant potential application value in the partial discharge detection of power distribution cabinets, providing a theoretical basis for its application in engineering practice.

## 1. Introduction

With the vigorous development of power systems, switchboard distribution cabinets have become widely deployed. They are of paramount importance in stable power supply, reasonable distribution, and the safe operation of equipment, and have a profound impact on trends in the power industry. Partial discharge (PD) is one of the main reasons for insulation failure in low-voltage switchgear, posing a serious threat to personal and equipment safety [1,2]. It may lead to fire accidents and even explosions [3,4]. Therefore, it is of great practical significance to study the detection method of PD in low-voltage distribution cabinets [5].

Researchers around the globe have focused on various physical phenomena in the discharge process for many years. Extensive works have been conducted on PD detection. Numerous PD detection approaches have been proposed recently, including the high-frequency current transformer detection approach (HFCT) [6], the ultra-high-frequency (UHF) approach [7,8], the ultrasonic detection approach [9,10,11], etc. These approaches are often used for on-site PD detection. Benefiting from its non-electrical detection characteristics, the ultrasonic detection approach has the advantage of being immune to electromagnetic interference and has long received much attention from scholars worldwide. As a result, a variety of ultrasonic detection methods have been successively proposed.

The implementation of ultrasonic methods usually relies on piezoelectric transducers (PZTs) to detect ultrasonic signals when PD occurs [3,12]. Insulation defects predominantly occur within the device. Consequently, the discharge signal undergoes significant attenuation by the time it reaches the outer shell, resulting in a weak effective signal. Piezoelectric ceramics exhibit a high dielectric constant and are susceptible to polarization. These materials possess multiphase characteristics, and the structure varies with temperature and electric field conditions. This variability leads to the limited anti-interference capability and reduced effective sensitivity of PZTs. By contrast, all-fiber-optical sensors possess advantages such as small size, light weight, corrosion resistance, and intrinsic safety. They can also be flexibly installed. Therefore, the all-fiber method has been proposed to overcome the above limitations.

The optical fiber sensing (OFS) unit converts the ultrasonic signals induced by PDs into optical signals, which avoids the conversion of electrical parameters during the sensing and signal transmission processes. Thus, extremely high sensitivity in the OFS unit could be achieved [6,13,14,15]. Scholars worldwide have proposed several optical fiber sensing methods for PD detection, such as the distributed optical fiber sensing system (DOFS) [16], fiber Bragg grating (FBG) detection method [17], Fabry–Perot interference-based detection method [18], Mach–Zehnder interference-based detection method [19,20], Michelson optical sensor detection method [21,22]. Among these, the Mach–Zehnder interference (MZI)-based detection method has become the preferred method for monitoring PD due to its high sensitivity, excellent temperature stability, and outstanding ability to detect small amplitude displacement and pressure changes.

The MZI-based optical fiber method was adopted in [23] to detect PD in oil–paper-insulated equipment. In this method, a mandrel-type single-mode fiber sensor with a diameter of 30 mm is utilized in an experiment bench. The PD sources are the parallel-plate electrode model and the needle-plate electrode model. The PD-generating device and the optical fiber sensor are placed in insulating oil. The PD detection comparative experiment is conducted between the optical fiber MZI and the traditional PZT sensor. The results have shown that the MZI-based PD detection method possesses higher sensitivity performance than that of the PZT-based PD detection method. Multilayer ceramics were used as the core rod for the optical fiber MZI sensor [24]. The experimental results indicate that the minimum fiber displacement that the optical fiber MZI can detect is 300 nm. The optical fiber MZI exhibits good performance in detecting ultrasonic signals. Relevant studies also show that the diameter of the optical fiber sensor will have a significant impact on the acoustic wave detection sensitivity [20]. Studies indicate that embedding a polarization-maintaining optical fiber within XLPE cable termination to construct an MZI system allows for the effective identification of PD signals as small as 196 pC, and the detection sensitivity of PDIV is increased by approximately 27.5% compared to that of high-frequency current transformers [25]. In addition, the power-function-curved mandrel structure (PMS) designed based on the acoustic black hole theory significantly improves the acoustic response performance of the external MZI sensor within the frequency range of 2–60 kHz. The sensitivity is increased by more than 50%, enabling more efficient long-distance PD acoustic wave sensing, which provides a new path for the engineering application of MZI in complex power scenarios [26].

Compared with the traditional ultrasonic detection method of PZTs (hereafter referred to as the PZT method), the MZI-based method has higher sensitivity and good electromagnetic isolation performance, which can effectively avoid the influence of electromagnetic interference on the signal. This paper proposes a PD detection method based on MZI for power distribution cabinet equipment (hereafter referred to as the MZI method), aiming to improve the detection accuracy of PD signals and their anti-interference capabilities in low-voltage distribution cabinets. To verify the effectiveness and feasibility of the proposed method in engineering applications, the MZI method is compared with the PZT method.

## 2. Mach–Zehnder Detection Principle

### 2.1. Mach–Zehnder Fiber Interference

The working mechanism of the fiber MZI is based on splitting the incident beam into two beams and propagating along different paths, one of which is affected by external stresses or vibrations such as stress waves, while the other remains unchanged. When the stress wave passes through the fiber, the geometric deformation of the fiber, such as stretching, compression, or bending, affects the optical path length of the propagating light. This results in a change in the phase difference between the two beams, which alters the interference pattern. Through the measurement of the change in light intensity, the relevant information of stress waves can be obtained.

The basic structure of the MZI consists of two optical splitters and two fiber arms. The input optical signal is split into two beams at the first splitter, which propagate along the two fiber arms. These two beams are remerged at the second beam splitter after moving along different propagation paths. When PD occurs, the resulting ultrasonic waves cause a small change in the refractive index in the fiber, which in turn causes a phase difference between the two beams along their propagation paths. The change in the phase difference is amplified by the interference phenomenon and converted to a change in light intensity, so as to realize the detection of PD.

The phase of the light wave propagating in the fiber is determined by the distance the light travels and the refractive index of the fiber. When the sound wave generated by PD passes through the fiber, it will cause small stresses and strains, which will change the refractive index of the fiber and then lead to phase changes. According to the interference principle, the phase difference Δ*ϕ* of the two beams can be described by

(1) $$\Delta \phi =\frac{2\pi L}{\lambda }\Delta n$$

where *L* is the length of the fiber, *λ* is the wavelength of light, and Δ*n* is the change in the refractive index caused by PD. The change in the phase difference eventually causes a change in the light intensity *I*, which can be expressed as

(2) $$I=I_{0}\left(1+\cos\left(\Delta \phi \right)\right)$$

It should be noted that Equation (2) assumes ideal polarization matching between the interfering beams. However, in practical scenarios, polarization mismatch between the reference arm and the sensing arm can lead to reduced interference visibility and signal attenuation. This effect is particularly pronounced in systems employing conventional single-mode fibers without utilizing polarization-maintaining mechanisms. Polarization effects must be considered when analyzing the stability and amplitude of interference signals.

Through the monitoring of the intensity change in the interference light, the tiny acoustic vibration triggered by PD can be detected in real time; thus, the occurrence and intensity of PD are captured.

MZIs are more resistant to electromagnetic interference than conventional piezoelectric sensors. This is because the fiber material is insensitive to electromagnetic fields, thus effectively avoiding the impact of the complex electromagnetic environment within the distribution cabinet on the detection of partial discharge signals. In addition, due to the high sensitivity of optical fibers, they are capable of detecting extremely feeble PD signals. This makes them particularly well suited for detection needs over long distances and in harsh environments.

### 2.2. Hilbert–Huang Transform (HHT)

PD acoustic waves are short-time pulse signals with short time intervals and low energy. The conventional time-domain waveform and Fourier analysis of PD acoustic waves are also very unsatisfactory. The HHT is an adaptive signal analysis method, which is used to analyze nonlinear and non-stationary signals. It consists of two main components: empirical mode decomposition (EMD) and Hilbert spectral analysis.

EMD is an adaptive signal decomposition method, which decomposes the signal into several simple oscillation components of intrinsic mode functions (IMFs). The purpose of EMD is to extract the IMFs from complex signals. Each modal component becomes a signal with local equilibrium, and its oscillation frequency varies over time. To decompose the steps of EMD, the local maxima and minima must first be extracted from the original signal. The local maxima and minima are then connected via interpolation to obtain the upper and lower envelopes of the signal, respectively. The average of the upper and lower envelopes is calculated, and the residual signal is obtained by subtracting this mean from the original signal. If the residual signal satisfies the symmetry of each extreme point as stated in the IMF condition and there is no trend over the whole signal, the signal is an IMF component. If the residual signal still contains a trend, the above steps are continued until all components in the signal have been decomposed into a number of IMFs. Once the signal has been decomposed into a number of IMFs, the Hilbert transform can then be applied to each IMF to obtain its instantaneous frequency and instantaneous amplitude. The Hilbert transform is expressed as

(3) $$\hat{x}(t)=H\left\{x(t)\right\}=\frac{1}{\pi }P\int_{-\infty }^{+\infty }\frac{x(\tau )}{t-\tau }d\tau$$

where *x*(*t*) is the original signal, $\hat{x}(t)$ is the imaginary part of the complex-valued signal obtained by applying the Hilbert transform to *x*(*t*), *t* is the point in time at which the computation is performed, and *P* indicates that the integral is the principal-valued integral and is used to deal with possible singularities. The signal can be expressed as a complex signal *z*(*t*) after Hilbert transformation.

(4) $$z(t)=x(t)+i\hat{x}(t)$$

According to the rules of complex calculation, the instantaneous amplitude *A*(*t*) and the instantaneous *ϕ*(*t*) phase can be calculated as follows:

(5) $$A(t)=\left|z(t)\right|=\sqrt{x{\left(t\right)}^{2}+\hat{x}{\left(t\right)}^{2}}$$

(6) $$\phi (t)=\arg(z(t))=\arctan\left(\frac{\hat{x}(t)}{x(t)}\right)$$

The instantaneous frequency represents how the frequency of a signal changes over time and is the derivative of the instantaneous phase with respect to time.

(7) $$f(t)=\frac{1}{2\pi }\frac{d\phi (t)}{dt}$$

Hilbert spectral analysis is used to obtain the instantaneous frequency and amplitude by applying the Hilbert transform to each IMF, and the characteristic of the signal’s spectrum changing over time is visualized, forming a time–frequency spectrum.

## 3. MZI-Based Optical Fiber Sensing Experiment Design for PD Detection

### 3.1. Fiber Ring Diameter and Acousto-Optic Effect

**Figure 1:**
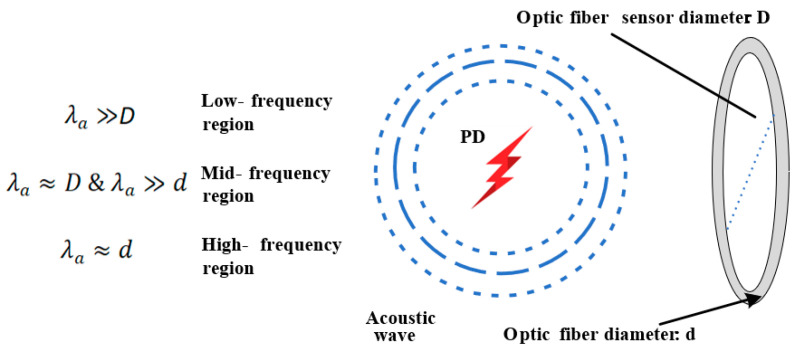
Schematic diagram of relationship between acousto-optic interaction and sensor diameter in fiber-optic sensor.

The fiber-optic ultrasonic sensor proposed in this paper uses a compact fiber ring structure with diameters of 50 mm, 80 mm, and 100 mm. Because of the relationship between the wavelength of the acoustic wave and the diameter of the fiber sensor, the acousto-optic effect of the fiber ring can be divided into three models. Model I: when the immediate acoustic wave is longer than the sensor diameter, the acousto-optic interaction is located in the low-frequency region, and the static pressure model can be used for analysis. Model II: When the wavelength of the acoustic wave is close to the diameter of the sensor and is significantly larger than that of the fiber, the acousto-optic interaction occurs in the mid-frequency region. Under such circumstances, apart from taking static pressure into account, the impact of the pressure gradient on the fiber sensor also needs to be considered. Model IIII: when the wavelength of the acoustic wave is equivalent to the diameter of the fiber, the acousto-optic interaction falls within the high-frequency region. The distribution of both the optical wave mode and the acoustic wave mode along the fiber should be considered in the analysis [25]. Figure 1 shows a schematic diagram of the relationship between acousto-optic interaction and sensor diameter in the fiber-optic sensor.

The ultrasonic PD detection bandwidth is generally 20 kHz to 300 kHz. Assume that the fiber coil is pasted on the cast steel shell of the distribution cabinet with aluminum foil tape. The propagation speed of a sound wave in cast steel is 5900 m/s, so the wave frequency of 20 kHz to 300 kHz corresponds to a wavelength of 295 mm to 19.7 mm, which is in the mid-frequency region of fiber acousto-optic interaction. The sound wave is not uniformly distributed on the fiber-optic sensor. The pressure distribution at any point in the fiber can be expressed as

(8) $$P=P_{c}e^{i\vec{K}\Delta \vec{r}}$$

where *P* is the pressure, *P_c_* is the pressure at *r_c_*, *K* is the wavenumber of the acoustic wave, and Δ*r* is the infinitesimal displacement position vector. The force *F*_0_ generated by the sound pressure action on the micro-element of the optical fiber sensor can be expressed as

(9) $$F_{0}=-2\pi AP\left[f\left(1-2P_{son}\right)J_{0}(\eta )-\eta J_{1}(\eta )\right]$$

where *A* is the cross-sectional area of the sensor, *P*_son_ is the Poisson’s ratio, $\eta =Kr_{c}sin\theta$, and *J*_0_ and *J*_1_ are the Bessel functions of the order 0 and 1, respectively. The external force causes the micro-element of the optical fiber sensor to generate a small displacement *μ*_0_ and therefore produces the corresponding inertial force, elastic force, and feedback force of the optical fiber sensor on the surrounding medium. The force balance equation is

(10) $$\Omega^{2}u_{0}\rho_{c}Ar_{c}\Delta \xi +F_{0}\frac{\Delta \xi }{2\pi }-AY_{g}u_{0}\frac{\Delta \xi }{r_{c}}+\Omega^{2}u_{0}\rho_{w}r_{c}\Delta \xi =0$$

where Ώ is the frequency of the sound wave, *μ*_0_ is the displacement generated by the action of sound pressure on the infinitesimal element of the sensing optical fiber, *r_c_* is the distance from the position of the infinitesimal element to the center of the sensor, Δ*ξ* is the divergence angle of the fiber-optic infinitesimal element relative to the center of the sensor, *F*_0_ is the driving force generated by the sound pressure, *Y*_g_ is the Young’s modulus, *ρ*_c_ is the density of the optical fiber, and *ρ_w_* is the density of the surrounding medium of the optical fiber. Therefore, the fiber ring diameter has a significant impact on the acousto-optic effect under the external acoustic wave, and the sensor performance can be optimized by adjusting the coil diameter.

### 3.2. Experimental Platform Design

**Figure 2:**
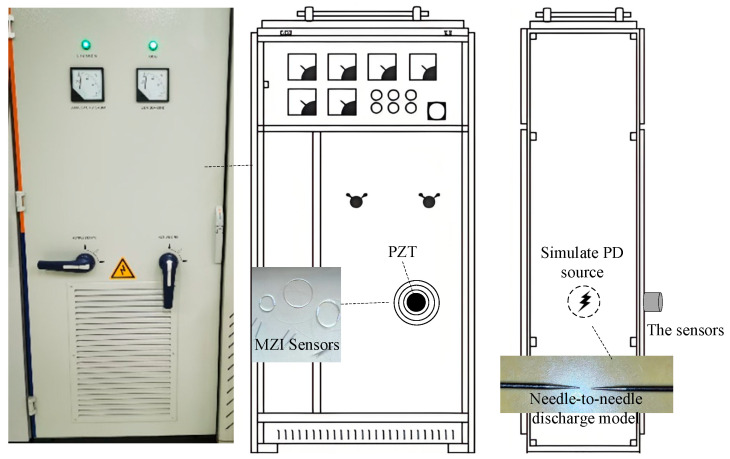
Experimental platform architecture.

This paper adopts a real distribution cabinet to carry out the PD detection experiment in the laboratory. Figure 2 illustrates a schematic diagram of the experimental platform. The electronic induction coil tip discharge device is used as the analog partial discharge power supply. The diameter of the pure tungsten electro-discharge needle is 1.5 mm, the tip length is 4 mm, and the pin-needle electrode spacing is 1 cm. To generate the discharge pulse, the output voltage is set as 12 kV. The PD source is overhead-mounted inside the cabinet, approximately 15 cm away from the cabinet door. The PZT sensor and three optical fiber sensors are pasted on the cabinet door, and the optical fiber length is 10 m. Each fiber-optic sensor is distributed in a concentric circle centered on the PZT sensor. The PZT has a built-in 40 db amplifier with a resonant frequency of 40 kHz, a frequency range of 30 kHz to 140 kHz, and a sensitivity of 120 dB.

### 3.3. MZI and Its Sensor Setup

**Figure 3:**
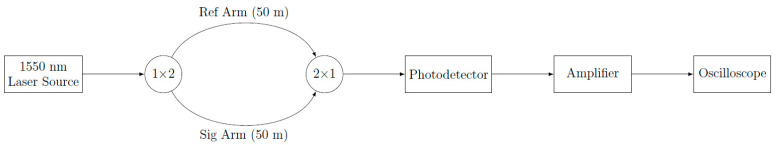
Structure diagram of optical fiber MZI PD detection device.

Figure 3 shows a structure diagram of the optical fiber MZI PD detection device. The MZI PD detection device consists of a laser with the output power of the 1550 nm narrow-linewidth type, two Corning 1550 nm single-mode optical fibers, two couplers with a 50:50 splitting ratio, a photodetector, an amplifier, and a Tektronix MDO34 oscilloscope.

The laser is 1.5 mW. Couplers 1 and 2 are both of the 3 dB 1 × 2 type. The optical fiber lengths of the reference and sensing arms are both 50 m, and the optical fiber sensing coil is located in the middle of the sensing arm. The sensor is arranged on the surface of the distribution cabinet box during sensing. In the experimental setup, the 50 m long reference arm is housed in a sealed enclosure lined with sound-absorbing foam to minimize the influence of external acoustic noise and environmental disturbances. This isolation measure is designed to ensure the phase stability of the reference optical path and enhance the overall quality of interferometric detection signals.

The photodetector is an InGaAs diode with a bandwidth of 1 GHz. Since the output signal of the photodetector is rather weak, in order to conduct a comparative analysis of the detected signal and the PZT signal, an external 10-time signal amplifier is connected. In the MZI system, the laser is divided into two parts: the first part enters the reference arm, and the second part enters the sensing arm. The optical fiber coil of the sensing arm is subject to disturbance from the outside world, which causes the phase change, and it is synthesized again with the reference-arm optical signal at coupler 2. The interference signal is received by the photodetector and converted into an electrical signal, which is recorded and analyzed by an oscilloscope with a 2.5 GHz sampling rate. In the experiment, three optical fiber sensors with large, medium, and small diameters are connected to the system’s optical path for PD detection. PD detection is also performed for the same discharge power source, and the PZT detection data are synchronously recorded for comparative analysis.

## 4. Experimental Analysis

### 4.1. Time–Frequency-Domain Analysis of Fiber-Optic MZI Sensing

**Figure 4:**
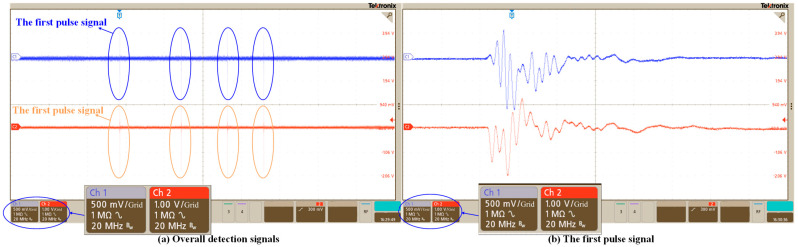
The time-domain signals of PD detected by the PZT (the red curve) and the fiber-optic MZI (the blue curve) with the center coil: (**a**) overall detection signals and (**b**) the first pulse signal.

Figure 4 shows the time-domain signals of partial discharge detected by the fiber-optic sensor MZI with a medium-sized coil of 8 cm in diameter and the PZT. The signal channel of the MZI is CH1, and the signal channel of the PZT is CH2. In Figure 4a, it is apparent that the MZI and PZT both clearly capture the sound wave pulse of the discharge power source, which is consistent in the time dimension. Four obvious pulses are captured in a time span of 4000 microseconds. The difference between the noise signal and local discharge pulse is obvious. Figure 4b shows that there is a difference in the initial detection time at the nanosecond level, which is caused by different detection techniques and sensor detection positions. The duration of the discharge acoustic pulse is very short, and the main part of the pulse is only about 400 ns. Therefore, if a Fourier analysis of the original signal is carried out, the frequency-domain information of the pulse wave will be submerged in the noise, and the relevant pulse frequency-domain features cannot be extracted.

**Figure 5:**
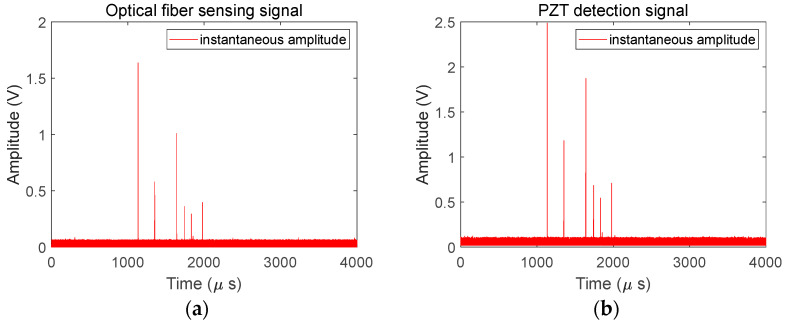
The instantaneous amplitude diagram after the application of the HHT to the detection signals from the medium-sized-coil MZI and PZT: (**a**) medium-sized-coil MZI and (**b**) PZT.

To detect and analyze PD acoustic pulse information more clearly, an HHT analysis of the original collected signal is carried out in this paper. Shown in Figure 5 is the instantaneous amplitude diagram after the application of the HHT to the detection signals from the medium-sized-coil MZI and PZT. To analyze the characteristics of the relationship between the amplitudes of the detection signals of the MZI and PZT, a set of signals with large differences in pulse amplitudes is selected for display. Figure 5 clearly shows the time-domain amplitude information of six pulse signals, which is easier to observe compared with that in Figure 4a. Through a comparison of Figure 5a,b, it can be found that the MZI and PZT also show consistency in detecting the maximum pulse values. The maximum values of the pulses detected by the MZI range from 0.294 V to 1.636 V, and correspondingly, the maximum values of the pulses detected by the PZT range from 0.544 V to 2.486 V.

**Figure 6:**
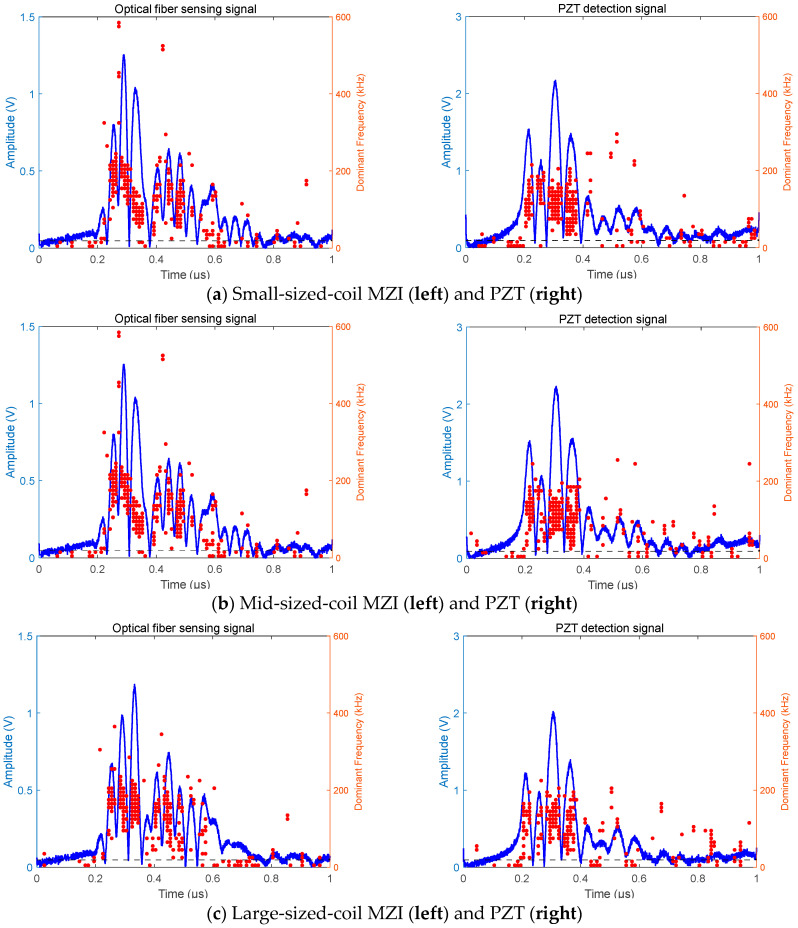
Comparison of the time–frequency-domain signals after the application of the HHT to single-pulse signals detected by the fiber-optic MZI and PZT (Blue curves: Amplitude. Red dots: Dominant frequency): (**a**) small-sized-coil MZI (**left**) and PZT (**right**), (**b**) mid-sized-coil MZI (**left**) and PZT (**right**), and (**c**) large-sized-coil MZI (**left**) and PZT (**right**).

Figure 6 shows the PD pulses detected by fiber-optic MZIs with coils of three different diameters and the corresponding PZT signals. A single pulse is selected for time–frequency-domain analysis using the HHT. The blue curve represents the superposition of the instantaneous amplitudes of the IMF after the HHT. The red dots denote the instantaneous frequencies of the IMF following the HHT. Meanwhile, the black dashed line at the bottom is set at 20 kHz, facilitating discrimination between ultrasonic and non-ultrasonic signals. According to IEC TS 62478 [27], ultrasound serves as a crucial indicator of PD. It is less susceptible to interference and is used as the primary detection target in PD detection via acoustic methods.

It can be found from the PZT pulse waveforms and the main frequency distribution in Figure 6 that the pulse characteristics of the three acoustic waves are similar, the interval from 0.2 to 0.4 microseconds is the main component of the pulse, that from 0.4 to 0.6 microseconds is the secondary component of the pulse, and the proportion of pulse components is low and the clutter is strong in the other time periods. The main frequency of the PZT signal for the three pulse waves shows that in the main pulse interval of 0.2 to 0.4 microseconds, the instantaneous frequencies of the pulses are mainly concentrated in the range of 20 kHz to 200 kHz. The pulse amplitude drops significantly between 0.4 and 0.6 microseconds, with its maximum value being less than half of that in the 0.2–0.4-microsecond interval. The pulse frequencies are more dispersed and sparser, rendering this interval of lower value for PD detection. In the remaining time periods, the signals are easily confused with noise and do not meet the requirements for PD detection judgment. Therefore, for PD pulse detection using the PZT, the pulses in the 0.2–0.4-microsecond time interval are the most valuable for reference.

A further comparative analysis of the time-domain diagrams of the pulses detected by the MZI and PZT in Figure 6 reveals that the main energy of the pulses is concentrated in the range of 0.2 to 0.4 microseconds. The difference lies in that the signal intensity of the MZI detection signal from 0.4 to 0.6 microseconds is stronger than that of the PZT during the same period, which is even more pronounced in the frequency domain. In the interval of 0.2 to 0.4 microseconds, the pulse frequencies detected by the MZI are higher than those of the PZT, with the main frequency distributed between 60 kHz and 260 kHz. Moreover, the pulse frequencies detected by fiber coil sensors with diameters of 5 cm and 8 cm exhibit a downward-sloping trend, containing more high-frequency components at the pulse front. In the 0.4–0.6-microsecond interval, the pulse frequencies are mainly concentrated in the range of 20 kHz to 220 kHz, which has values large enough for PD judgment. Given the extremely short duration of PD acoustic pulses, increasing the effective detection time length of PD pulses and frequency-domain information is of great significance, as it can improve detection accuracy and prevent misjudgment. Meanwhile, the frequency band detected by the MZI is wider than that of the PZT.

### 4.2. The Impact of Sensor Coil Diameter on PD Detection

The diameter of the fiber-optic coil will affect the sensitivity of the fiber MZI to the acoustic detection of different frequencies. The high-frequency acoustic component is more sensitive to the size of the fiber-optic coil. The diameter of the fiber-optic sensing coil can be appropriately reduced to increase high-frequency detection sensitivity. This study employs the Corning SMF28e 1550 nm single-mode optical fiber, exhibiting typical bending losses of approximately 0.5 dB per turn at a 15 mm bending radius, 0.03 dB per turn at 20 mm, and 0.002 dB per turn at 30 mm. The impact of bending loss requires careful consideration in small-diameter-sensor design.

To investigate the influence of the diameter of the fiber coil on PD detection inside the distribution cabinet, three 10 m length fiber coils with diameters of 5 cm, 8 cm, and 10 cm are designed as sensors for the MZI. According to the main frequency comparison of MZI detection signals in Figure 5, the performance of the fiber sensors with 5 cm and 8 cm diameter coils is significantly better than that of 10 cm diameter coils in PD acoustic wave signal detection above 200 kHz, and the high-frequency components in the pulse are more abundant.

**Figure 7:**
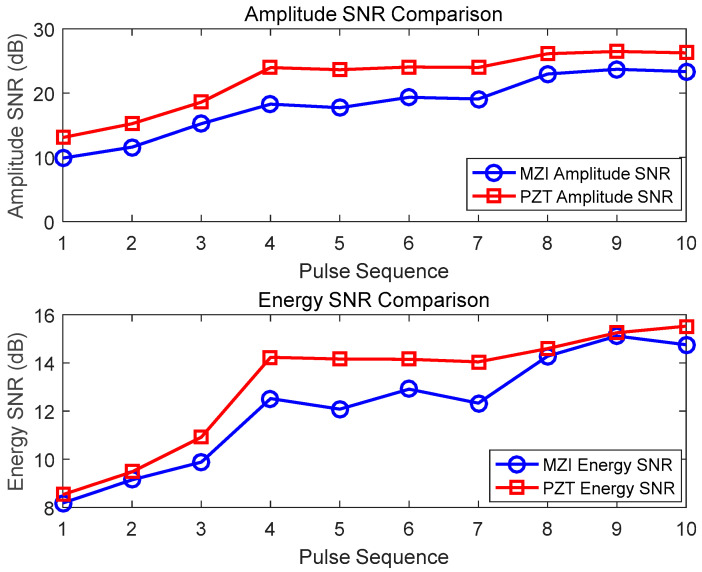
Detection results of 5 cm diameter optical fiber sensors and PZT for partial discharge pulses.

Figure 7 indicates the SNR data of the PD signals detected 10 times by the MZI method with a 5 cm optical fiber coil and the PZT method. For the amplitude SNR, the values of the MZI span from 9.88 to 23.71, with an average of 17.32. In contrast, the corresponding values of the PZT range from 13.08 to 26.49, averaging at 21.37. Regarding the energy SNR, the MZI values lie between 8.18 and 15.11, with an average of 12.16, while the PZT values are in the range of 8.53 to 15.52, with an average of 13.77. Overall, both the MZI and the PZT display positive correlation trends. The 5 cm optical fiber coil shows an acceptable response in the energy dimension. However, it generally has a lower SNR compared to the PZT, suggesting that its detection performance is somewhat limited.

**Figure 8:**
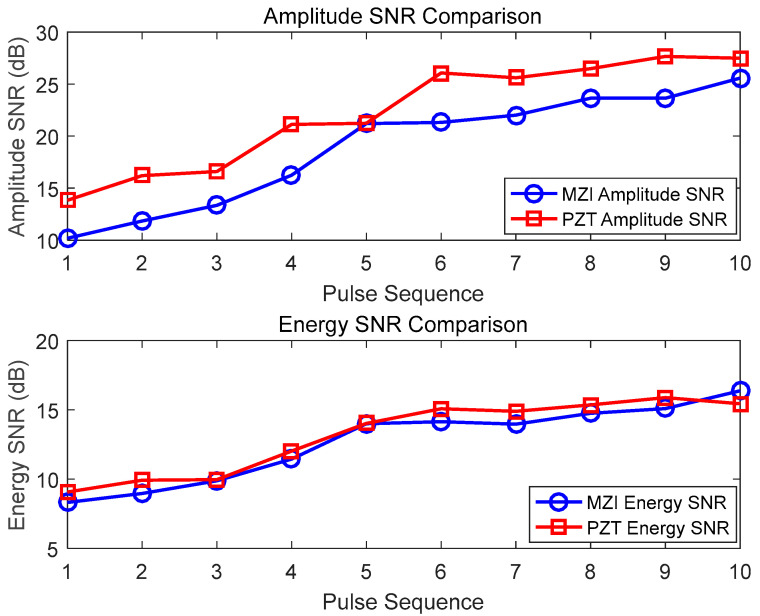
Detection results of 8 cm diameter optical fiber sensors and PZT for partial discharge pulses.

Figure 8 shows the SNR data of the PD signals detected 10 times by the MZI method with an 8 cm optical fiber coil and the PZT method. For the MZI, the amplitude SNR ranges from 10.18 to 25.58, with an average of 18.60. In contrast, the corresponding amplitude SNR of the PZT ranges from 13.82 to 27.67, with an average of 21.86. The energy SNR of the MZI is in the range of 8.32 to 16.37, with an average of 13.59, while the corresponding energy SNR of the PZT ranges from 9.07 to 15.88, with an average of 13.60. It can be observed that the amplitude SNR of the 8 cm coil MZI approaches the level of the PZT, and the energy SNR is basically on par with that of the PZT. Some of the data points of the MZI’s energy SNR are even slightly higher than those of the PZT. This indicates that a sensor of this size has excellent capabilities for detecting PD acoustic waves.

**Figure 9:**
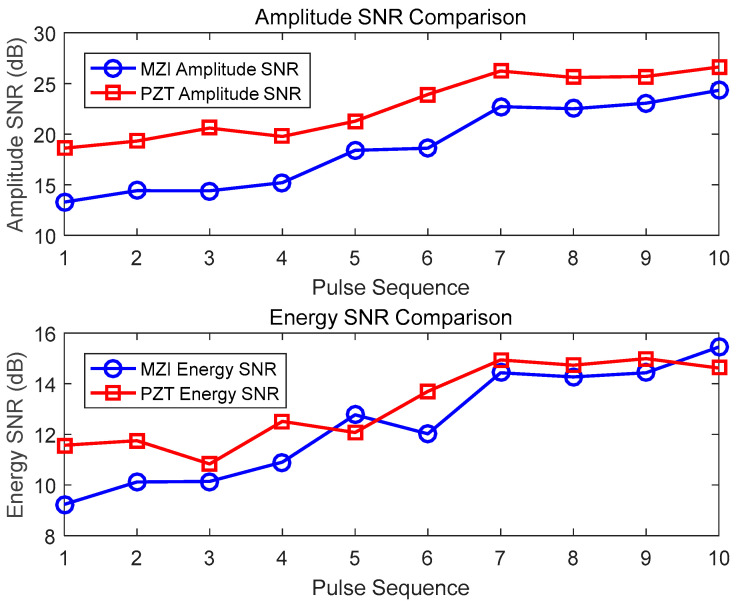
Detection results of 10 cm diameter optical fiber sensors and PZT for partial discharge pulses.

Figure 9 shows the SNR data of 10 PD signal detections using the MZI method with a 10 cm optical fiber coil and the PZT method. The amplitude SNR of the MZI ranges from 13.29 to 24.33, with an average of 18.17; the amplitude SNR of the PZT ranges from 18.61 to 26.63, with an average of 23.12. The energy SNR of the MZI ranges from 9.23 to 15.45, with an average of 13.08; the corresponding energy SNR of the PZT ranges from 10.83 to 14.99, with an average of 13.45. Although the SNR of some of the data points of the 10 cm coil demonstrates good performance, there is significant fluctuation, and its sensitivity and stability are slightly inferior to those of the 8 cm coil.

Therefore, in a comprehensive analysis of the average values of the amplitude and energy SNRs of the three types of optical fiber coils, the optical fiber sensor with a diameter of 8 cm shows the best performance in the experiment. While maintaining a good amplitude response, the MZI method with an 8 cm optical fiber coil achieves an effect equivalent to or even better than that of the PZT sensor in terms of the energy SNR, combining both sensitivity and stability. Since there are few samples of the sensor diameter, there is still room for further optimization.

## 5. Conclusions

This paper proposes three fiber-optic MZI sensing systems for PD detection with different-fiber-coil-diameter sensors, and a PD detection experimental platform with a real distribution cabinet is developed. The PD detection experiment is carried out in the laboratory, and it is verified by comparative analysis with commercial PZT sensors. Through the HHT analysis of the collected data, the following conclusions are obtained:

(1) The fiber MZI technology can be used to detect the PD of the distribution cabinet, and the amplitude of the PD acoustic wave in the time domain detected by the fiber MZI is consistent with the PZT.
(2) The length of the PD acoustic pulse in the distribution cabinet is 0.4 microseconds, and the main energy is concentrated in the first 0.2 microseconds of the pulse. Based on the distribution of ultrasonic signal and PD determination requirements, the effective time length of a single PD pulse detected by the fiber MZI is 0.4 microseconds, and the effective pulse length of a single PD pulse detected by the PZT is 0.2 microseconds. Thus, the frequency detection range of the fiber MZI is wider than that of the PZT.
(3) The sensing characteristics of optical fiber coil sensors with diameters of 5 cm, 8 cm, and 10 cm, all with a length of 10 m, are compared. The results show that the higher the acoustic wave frequency, the more sensitive the response to the size of the coil diameter. In a comprehensive analysis of the frequency characteristics, the SNR of the pulse amplitude, and the SNR of the pulse energy, the optical fiber sensor with a diameter of 8 cm in this paper performs the best.

In summary, the fiber MZI shows its advantages in the range of the PD acoustic wave detection frequency band, the effective time length of PD acoustic pulse detection, anti-electromagnetic interference, and layout flexibility. This paper further consolidates a theoretical foundation for the engineering application of optical fiber sensing technology in the field of PD. More simulation experiments will be conducted in the future to verify the effectiveness of this method in more challenging PD scenarios.

## Data Availability

The data presented in this study are available on request from the corresponding author.
